# Adapting MRI as a clinical outcome measure for a facioscapulohumeral muscular dystrophy trial of prednisone and tacrolimus: case report

**DOI:** 10.1186/s12891-020-03910-1

**Published:** 2021-01-09

**Authors:** Leo H. Wang, Laura M. Johnstone, Michael Bindschadler, Stephen J. Tapscott, Seth D. Friedman

**Affiliations:** 1 Department of Neurology, University of Washington, Seattle, Washington USA; 2 University of Washington Medical Center, Box 356465, 1959 NE Pacific Street, Seattle, WA USA; 3 Department of Rehabilitative Medicine, University of Washington, Seattle, Washington USA; 4 Radiology Clinical Research Imaging Core/Neurology, Seattle Children’s Hospital, Seattle, Washington USA; 5 Human Biology Division, Fred Hutchinson Research Center, Seattle, Washington USA; 6 Radiology Clinical Research Imaging Core/Improvement and Innovation, Seattle Children’s Hospital, Seattle, Washington USA

**Keywords:** All neuromuscular disease, Muscle disease, Facioscapulohumeral muscular dystrophy (FSHD), Outcome measures, Case report

## Abstract

**Background:**

Facioscapulohumeral muscular dystrophy (FSHD) is a patchy and slowly progressive disease of skeletal muscle. MRI short tau inversion recovery (STIR) sequences of patient muscles often show increased hyperintensity that is hypothesized to be associated with inflammation. This is supported by the presence of inflammatory changes on biopsies of STIR-positive muscles. We hypothesized that the STIR positivity would normalize with targeted immunosuppressive therapy.

**Case presentation:**

45-year-old male with FSHD type 1 was treated with 12 weeks of immunosuppressive therapy, tacrolimus and prednisone. Tacrolimus was treated to a goal serum trough of > 5 ng/mL and prednisone was tapered every month. Quantitative strength exam, functional outcome measures, and muscle MRI were performed at baseline, week 6, and week 12. The patient reported subjective worsening as reflected in quantitative strength exam. The MRI STIR signal was slightly increased from 0.02 to 0.03 of total muscle; while the T1 fat fraction was stable. Functional outcome measures also were stable.

**Conclusions:**

Immunosuppressive therapy in refractive autoimmune myopathy in other contexts has been shown to reverse STIR signal hyperintensity, however this treatment did not reverse STIR signal in this patient with FSHD. In fact, STIR signal slightly increased throughout the treatment period. This is the first study of using MRI STIR and T1 fat fraction to follow treatment effect in FSHD. We find that STIR might not be a dynamic marker for suppressing inflammation in FSHD.

## Background

Facioscapulohumeral muscular dystrophy (FSHD) is a patchy and slowly progressive disease of skeletal muscle [1]. It is one of the most common muscular dystrophies that is the result of a toxic gain-of-function from de-repression of the *DUX4* gene that is not normally expressed in skeletal muscle. The muscle histopathology underlying the disease is also variable, reflecting the patchy clinical involvement. Inflammation may be present in a subset of biopsies [2–4]. Given the intensity of the inflammation in some biopsies, pathologists have at times interpreted the histopathology as representing an immune-mediated disease such as polymyositis [2]. The inflammatory cells are endomysial, surrounding intact fibers, and often perivascular.

Multiple studies have assessed muscle MRI in patients with FSHD focusing on determining muscles to follow in a clinical trial either by imaging or muscle biopsy (looking for DUX4-downstream signature markers) [5–10]. MRI STIR sequences null the fat signal and highlight elevated free water signal. STIR hyperintensities indicate muscle edema, a feature associated with acute changes such as inflammation, trauma, metabolic derangements, or infection [11]. Two studies, Frisullo et al. and Tasca et al., performed biopsies on muscles with STIR hyperintensities and found 5/5 biopsies with endomysial CD8+ cells, perivascular CD4+ T-cells and CD68+ cells [12, 13]. Frisullo et al. found increased percentage of circulating CD8+pSTAT1+, CD8+T-bet+, and CD14+pSTAT1+ cells in 14/25 peripheral blood samples of FSHD patients with STIR hyperintensities, a significant increase when compared to FSHD patients without STIR hyperintensities and healthy controls. In the Seattle Wellstone FSHD study of 36 patients with MRI-guided biopsies, we found that MRI STIR positivity was associated with inflammation or active myopathy in ~ 70% of the muscle biopsies versus 25% of the STIR negative muscles [10]. A most recent paper by Dahlqvist et al. found that STIR-positive muscles indicating muscle inflammation were associated with faster muscle degradation [14]. Therefore, our hypothesis was that if STIR hyperintensities represent active inflammation, these abnormalities would normalize with targeted therapy.

We chose a combination of prednisone because of its fast onset and effect on both B and T cells; and tacrolimus for its effects on T cells. This multi-agent approach was selected given that a prior, open-label, 12-week trial of immunosuppression with prednisone (1.5 mg/kg/day) in eight FSHD patients failed to show improvement in muscle strength as assessed by manual muscle testing or maximum voluntary isometric contraction testing [15]. What was not evaluated in that study was MRI imaging which could certainly be more sensitive and specific than strength.

In refractory inflammatory myopathies, the use of a calcineurin inhibitor offers effective treatment [16]. STIR signal has been reported to be reversed in small case series of patients with inflammatory myositis [17] also in a case of TNF receptor-associated periodic syndrome [18]. We selected a three-month treatment duration similar to the duration of treatment trials for inflammatory myopathies to test our hypothesis.

## Case presentation

**Fig. 1:**
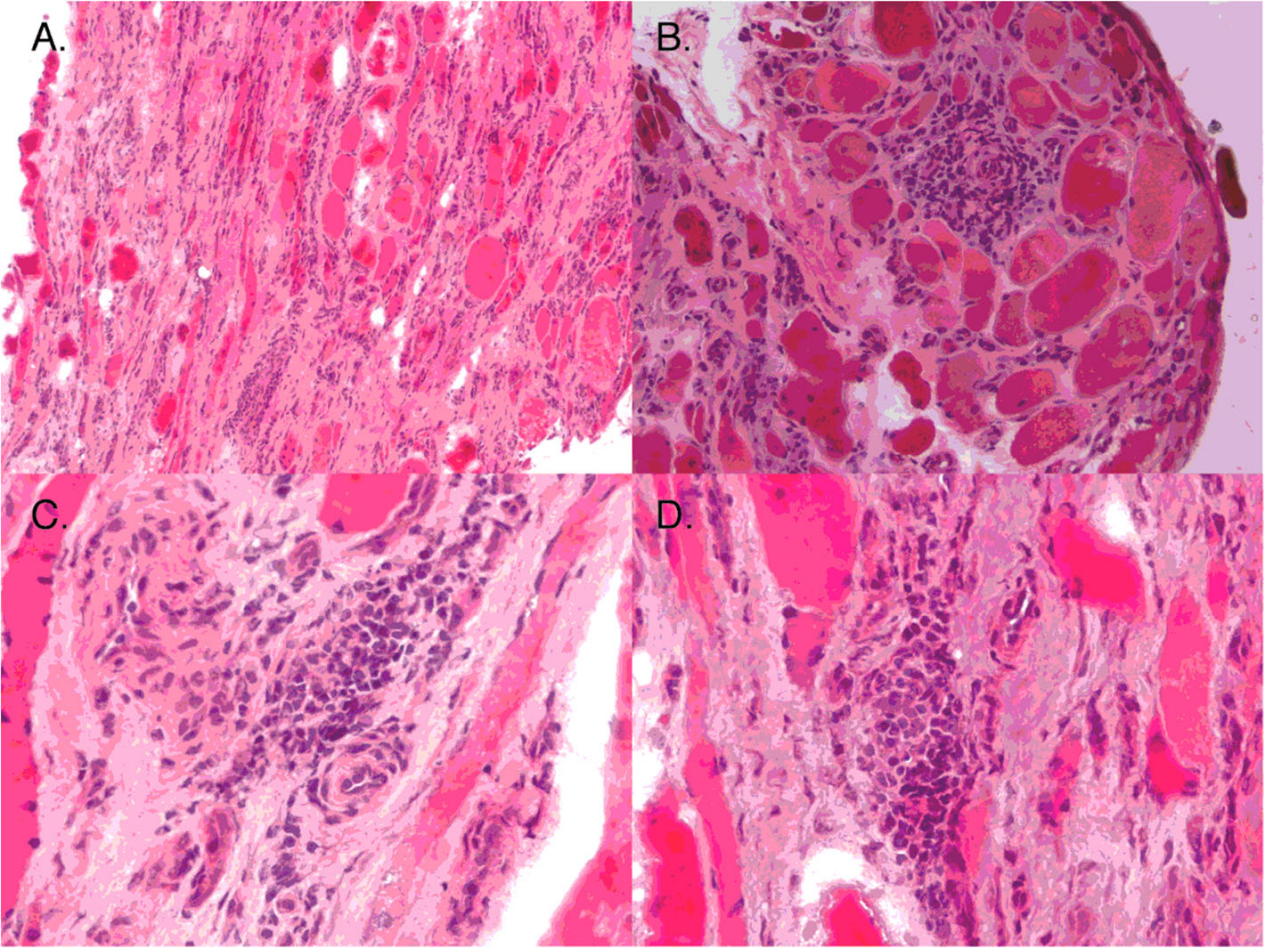
Muscle histopathology of left medial gastrocnemius. Muscle histopathology showed a chronic myopathy with severe myofiber size variability, necrosis, and increased endomysial inflammation (**a**); also present was perivascular inflammation (**b**-**c**). Courtesy of Dr. Rabi Tawil

The subject was a 45-year-old male with history of diabetes mellitus type 2 and FSHD type 1 (with clinical severity score of 2 and nine D4Z4 repeats) who first noticed symptoms at age 19, when he did 200 pull-ups as part of the Air Force Academy and thereafter lost the ability to do further pull ups. He also noticed lordosis while walking and attributed that to injury on an obstacle course. At the age 35, he realized that he could no longer set as a semi-pro volleyball player. At the age 39, after prolonged biking for a triathlon, he could no longer walk downstairs as he had problems stopping his forward progress. He then noticed that his arms got worse. He denied facial weakness but stated that his face gets tired and has been told that he never smiles. He denied problems with sucking on a straw or whistling. On exam, he demonstrated strong eyelid and lip closure, and cheek puff. Tongue and sternocleidomastoids were strong. His appendicular strength was intact, with the patient able to do deep knee bend but having a hard time walking on toes and heels. As part of the Seattle Wellstone study, an MRI showed STIR positivity in his left medial gastrocnemius and a biopsy of that muscle showed a chronic myopathy with severe myofiber size variability, necrosis, and increased endomysial inflammation (Fig. 1a); also present was perivascular inflammation (Fig. 1b-c).

After informed consent was obtained, the patient was treated for 3 months with an immunosuppressive regimen of tacrolimus (2.5–3 mg twice per day for goal trough 4 ng/dL) and prednisone (40 mg per day for the first month, 20 mg per day for the second month, and then 10 mg per day for the third month). Quantitative strength exam, functional outcome measures, and muscle MRI were performed at baseline, week 6, and week 12.

All MRI examinations were performed on a 3 T Philips Ingenia scanner. Sequences were acquired using flexible array coils to cover the pelvis down to the ankles in multiple stations (more to cover anatomy with thinner slices) with the following parameters in the axial acquisition plane: T1-weighted DIXON (Fast Field Echo [FFE] readout, four anatomical blocks [stations]: TE=1.35/2.58 ms, TR=shortest [example 4.12 ms], field-of-view [FOV] phase=300, FOV read=max, matrix=448 × 272, in-plane resolution=1.6 × 1.6 mm [thigh], 1.1 × 1.1 mm [calf], slice thickness=3 mm), STIR (three stations: TE=42 ms, TR=5277 ms, FOV phase=410, FOV read=max, matrix=448 × 260, in-plane resolution=1.1 × 1.1 mm, slice thickness=9 mm, IR delay=220 ms), and T2-weighted DIXON (three stations: TE=97/97 ms [phase difference], TR=5570 ms, FOV phase=410, FOV read=max, matrix=448 × 272, in-plane resolution=1.1 × 1.1 mm, slice thickness=9 mm). Total exam time was approximately 40 min equating to ~ 4 min per sequence (four stations T1 and three for STIR and T2 DIXON).

**Fig. 2:**
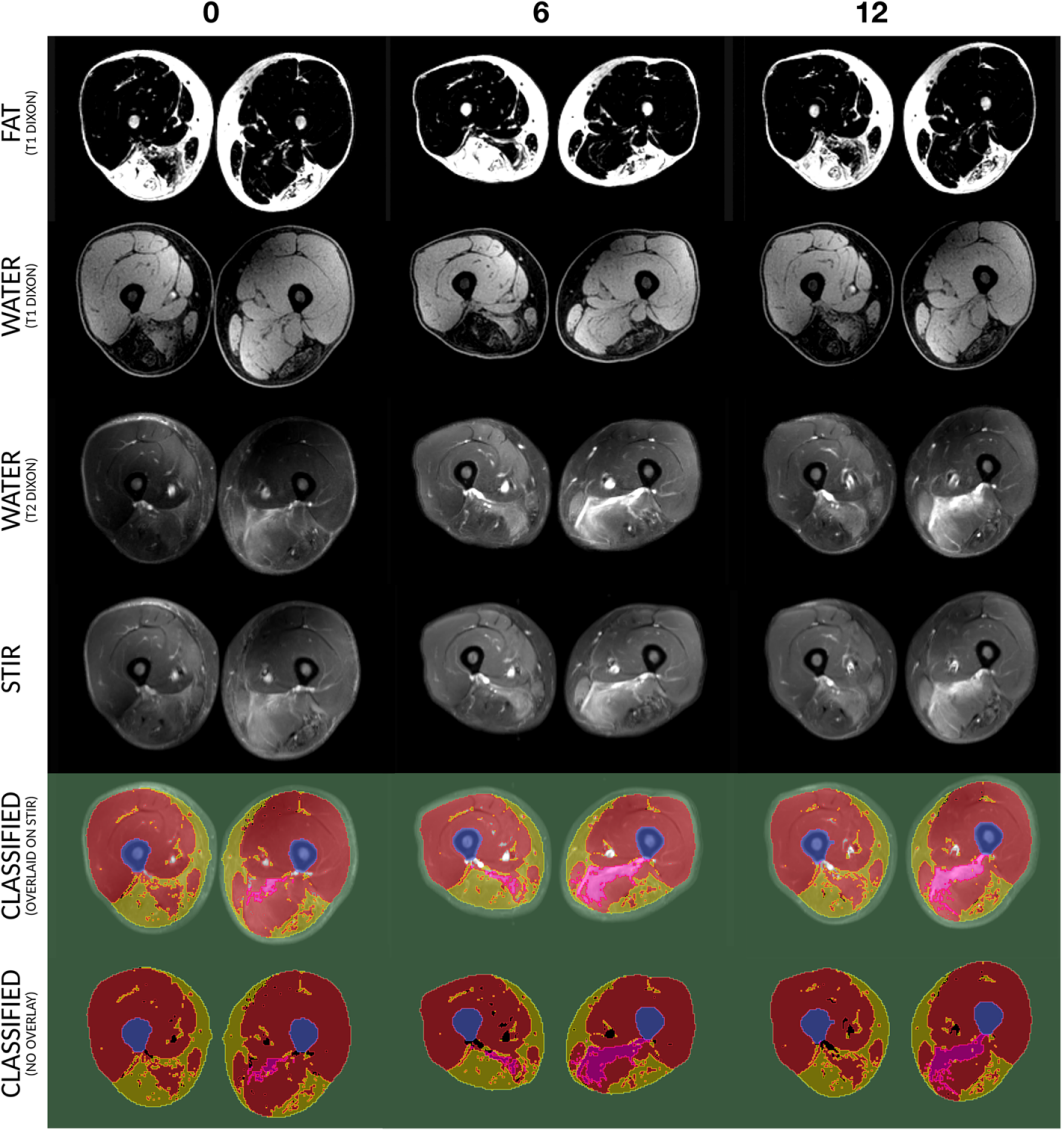
Vector Classification Example: From top, fat (T1 DIXON), water (T1 DIXON), water (T2 DIXON), and STIR images are shown from the same anatomical locations. Note the overlap between T2-weighted water measures and STIR+ signal. The vector segmentation overlaid on the STIR images follows, demonstrating the close correspondence between classes of air and subcutaneous tissue (green), bone (blue), muscle (red), fat (yellow), and STIR+ signal (pink). In the raw segmentation without overlay (bottom), black pixels are edited large vessels

MRI data were imported into Slicer (http://www.slicer.org/, [19]) for processing. The algorithm developed creates a registered image set across the different image types. When you select a pixel within a region, for example a fatty replaced muscle compartment, across the registered images you get back a vector of voxel values, one value for each registered image at that spatial location. Since voxel values depend on the tissue type and on the MRI signal sequence, voxels with similar value vectors are likely to represent the same tissue type. The steps to select model classes and generate hard classified images is described in more detail below. Images underwent preprocessing steps: 1) stitching to join the separate series into a contiguous volume, 2) interpolation of all series to the highest resolution data (T1 DIXON), and 3) separate series intensity normalization and inhomogeneity correction. Next, data were exported for segmentation in MATLAB (Mathworks, Natick MA), with the time-points concatenated into a common block and then seeds were selected in the sum exams for the four primary classes of interest (fat, muscle, STIR+, bone). From these samples, gold-standard voxel value vectors were generated and the images classified by voxel vector similarity (least squared vector difference). To ensure that bone and sub-cutaneous fat were not included in volume measurements, both classes were expanded (digital dilation) by 5 and 4 pixels respectively. This has the practical result of removing the subcutaneous fat ring and ensuring that the bone mask is liberal enough to ensure that it is not being incorporated into muscle measures. As a last step, since bladder and vasculature appear as STIR bright features, these were removed the final segmentations by a rater blinded to time-point. This vector classification approach yielded excellent overlap between the multi-modality images for major tissue classes of interest (fat, muscle [normal-appearing], and STIR+ regions) as can be seen in Fig. 2. Summary volumes were computed for evaluating change with treatment.

**Fig. 3:**
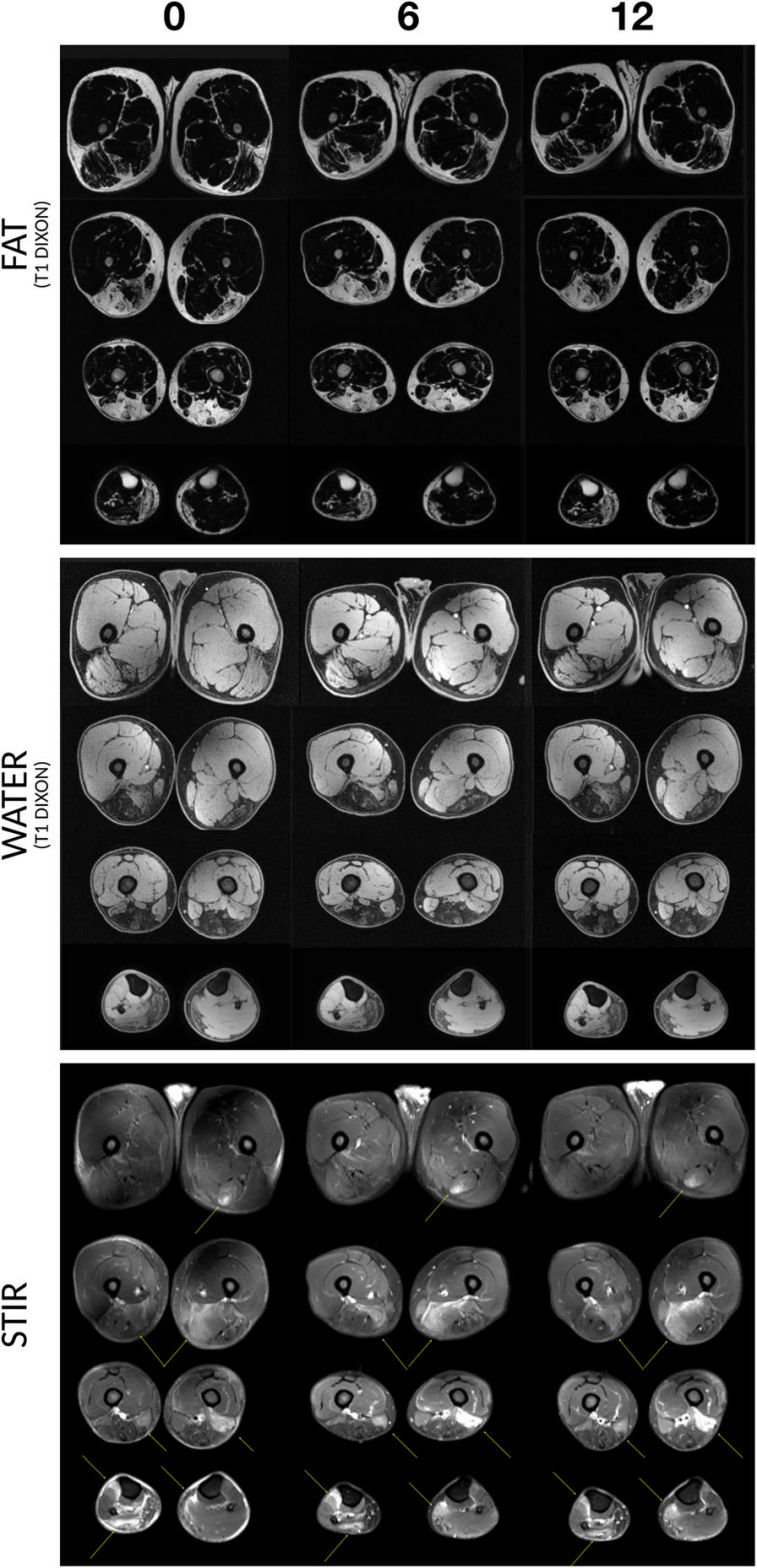
STIR and T1 MRI of lower extremity over the treatment period (6 and 12 weeks). The fat and water images derived from the T1 DIXON scan are shown at top and middle throughout the leg volumes across the study interval. In the bottom panel, regions with STIR+ signal are shown with arrows. The STIR+ signal persists at the two treatment time-points

There was no reversal of the abnormal STIR signal elevations seen at baseline by visual evaluation or by the volumes derived. Numerically, there was a slight increase in the fraction of muscle with STIR positivity from 0.02 at baseline to 0.03 at week 12 (Table 1). Data were expressed STIR/total at each time-point to account for weight changes. The stability of this biomarker and its potential to increase over the time-course can be seen in Fig. 3, with arrows demarcating areas of STIR+ features seen across the thigh and calf. Fat fraction assessed in bilateral legs showed no increase between baseline and at the end of the treatment period (12 weeks) with fat fraction at 0.27.

**Table 1:** MRI characteristics over 12 weeks of treatment

	Baseline	6 weeks	12 weeks
STIR hyperintensity/muscle + STIR positivity	0.02	0.03	0.03
Fat fraction	0.27	0.28	0.27

Quantitative strength examination of the lower extremity by quantitative myometry showed mild decrease in bilateral knee flexion and ankle dorsiflexion strength (Table 2). Functional outcome measures showed no clinically significant changes (Table 2).

**Table 2:** Quantitative myometry (in Newtons) and functional outcome measures

	Baseline	6 weeks	12 weeks
Knee flexion (right, N.)	22	13	13
Knee flexion (left)	62	40	36
Knee extension (right)	209	182	200
Knee extension (left)	227	218	209
Ankle dorsiflexion (right)	76	62	58
Ankle dorsiflexion (left)	89	62	36
6-min walk test (m)	600	580	596
Time up and go (sec)	5	5	5
Self-selected gait speed (sec)	4	3	3
Go 30 ft (sec)	4	4	4
Ascending stairs (sec)	2	2	2
Descending stairs (sec)	2	2	2
Sit to stand (sec)	1	1	1

The patient lost 9 kg during the study from a baseline weight of 104 kg. He reported no significant side-effect of the medications. Labs showed persistent hyperglycemia with hemoglobin A1c 6.6% at baseline and 8.8% at week 12. Tacrolimus trough levels were consistently therapeutic between 7.8 and 10.7 ng/mL. Renal function, potassium, magnesium, and phosphate levels were unaffected throughout the study.

## Discussion and conclusions

Despite literature data (Tasca, Frisullo, Dahlqvist, our prior work [10, 12–14]) suggesting that STIR+ features may be primarily related to inflammation, we saw no change in signal intensity during a 12-week treatment trial with tacrolimus and prednisone. If we assume that STIR-positivity is primarily related to inflammation, then there may be a chance that the inflammation was not reversed with our regimen and that a more stringent regimen was required; however, we were using therapeutic doses of tacrolimus recommended for the treatment of inflammatory myopathies [16]; and therefore this is unlikely. Notable is a study [18] where complete resolution in STIR elevations did occur with equivalent immunosuppressive agents and time-course in a different muscle disease. However, this must be interpreted cautiously in our study, as while the patient clearly had inflammation on muscle biopsy prior to the study, we do not have a follow-up muscle biopsy to prove that our immunosuppressive regimen reduced the inflammation.

If we assume that our immunosuppressive regimen was therapeutic, then our findings support the idea that the STIR hyperintensity does not exclusively depend on active inflammation. This is a conclusion that we favor. While it could be envisioned that small inflammatory components would be modulated by our immunosuppressive regimen, the bulk of the abnormality remained stable and this perhaps reflects other pathological features such as edema, vasculature changes, fibrosis, and macroscopic features of cell death and reorganization. Such inflammatory mimicry in STIR signal is found in a broad range of diseases [20].

While we can debate the significance of the STIR signal, the immunosuppressive regimen of tacrolimus and prednisone did not improve strength as our patient subjectively reported worsened strength. This was borne out in the quantitative myometry. While it was unclear whether the changes in quantitative myometry were clinically significant, within error of measurement, or reflective of a volitional component, the patient’s strength measurements reflected his subjective experience during the study. There were other measurements that were stable throughout the study, such as distance walked in 6 min and the time in the time-up-and-go tests. Furthermore, his total T1 fat fraction of the leg muscles measured was unchanged suggesting that there was no progression in fatty replacement of muscle—a measure associated with increasing functional disability.

The inability of tacrolimus and prednisone to reverse the significant STIR hyperintensities on this patient’s muscle MRI, suggest that this regimen most likely will not succeed in reversing the STIR-positive signal in a larger study. We can also conclude that the short course of the immunosuppressive regimen of tacrolimus and prednisone did not improve the strength manifestations of FSHD. While it could always be argued that larger samples and/or longer studies are needed to generalize a single-subject result, the regimen and study design employed is not without risk. Our study shows the importance of objective outcome measures such as T1 fat fraction, representative of fibrofatty replacement, and functional outcome measure, as useful indices to infer the ever-hopeful treatment response.

## Data Availability

The datasets used and/or analysed during the current study are available from the corresponding author on reasonable request.

## Abbreviations

FSHD: Facioscapulohumeral muscular dystrophy
MRI: Magnetic resonance imaging
STIR: short tau inversion recovery
